# Application of Biomedical Microspheres in Wound Healing

**DOI:** 10.3390/ijms24087319

**Published:** 2023-04-15

**Authors:** Caihong Yang, Zhikun Zhang, Lu Gan, Lexiang Zhang, Lei Yang, Pan Wu

**Affiliations:** 1 State Key Laboratory of Targeting Oncology, National Center for International Research of Bio-Targeting Theranostics, Guangxi Key Laboratory of Bio-Targeting Theranostics, Collaborative Innovation Center for Targeting Tumor Diagnosis and Therapy, Guangxi Medical University, Nanning 530021, China; 2 Wenzhou Institute, University of Chinese Academy of Sciences, Wenzhou 325001, China; 3 School of Pharmacy, Guangxi Medical University, Nanning 530021, China

**Keywords:** microspheres, wound healing, biomaterials, drug release, tissue engineering

## Abstract

Tissue injury, one of the most common traumatic injuries in daily life, easily leads to secondary wound infections. To promote wound healing and reduce scarring, various kinds of wound dressings, such as gauze, bandages, sponges, patches, and microspheres, have been developed for wound healing. Among them, microsphere-based tissue dressings have attracted increasing attention due to the advantage of easy to fabricate, excellent physicochemical performance and superior drug release ability. In this review, we first introduced the common methods for microspheres preparation, such as emulsification-solvent method, electrospray method, microfluidic technology as well as phase separation methods. Next, we summarized the common biomaterials for the fabrication of the microspheres including natural polymers and synthetic polymers. Then, we presented the application of the various microspheres from different processing methods in wound healing and other applications. Finally, we analyzed the limitations and discussed the future development direction of microspheres in the future.

## 1. Introduction

Wound healing is a complex, cascading, and finely organized predetermined process that aims to repair the physiological process of disruption of skin continuity due to exogenous causes (e.g., trauma, compression and burns) or endogenous causes (e.g., metabolic or vascular disease) [1,2,3]. These cascade processes include cell migration, secretion of cytokines, interleukins, growth factors, and secretion of extracellular matrix proteins, as well as a series of complementary repair processes [4,5,6]. Finally, the missing tissue is replenished through the synergistic effects of a series of molecular, biochemical, and cellular processes, which can complete the anatomical reconstruction of the tissue and restore tissue integrity [7,8,9]. The wounds can develop into chronic wounds, such as diabetic wounds and infected wounds, which are characterized by hypoxia and infection. To promote wound healing and reduce scarring, various wound dressings, such as gauze, bandages, sponges, patches, and microspheres, have been developed. Compared with the microspheres, after the gauze, bandages, patches, and sponges are used for wounds, the condition of wounds is not easy to observe due to shaped formulation and non-breathable state. They may become an obstacle in the process of wound contraction due to their limitation in the volume [10]. In addition, these dressings need to be pressed on the bleeding wounds because of the lack of bio-adhesion, thus they cannot be used for non-compressible bleeding wounds [11]. However, among these dressings, the microspheres can flexibly adapt to the wound area and promote wound healing due to its various advantageous properties such as flexible applicability and good biocompatibility.

Microspheres have been adequate to deliver the small molecule drugs, gene vaccines, stem cell and peptide proteins [12,13,14]. Microspheres can be embedded with solid or liquid drugs to protect them from destruction, improve stability and reduce irritation [15]. The key to the preparation of microspheres is not only to maintain the original activity of the drug, but also to have a high drug encapsulation rate, uniform particle size of the microsphere, and good reproducibility of the preparation process [16]. This requires the ability to not only understand the chemical and physical properties of the drug, but also be familiar with the properties and preparation techniques of commonly used materials when preparing microspheres. Moreover, the degeneration of microspheres derived from different materials affects the drugs release rate. The faster the degradation of the microspheres, the faster the release of the drug [17]. In addition, different microspheres prepared by different methods and materials have different properties and application. Thus, different methods and materials should be discussed and analyzed. Therefore, it has important theoretical significance and practical value that polymer microspheres with uniform diameter distribution and good monodispersity are prepared by selecting suitable methods and materials.

**Figure 1:**
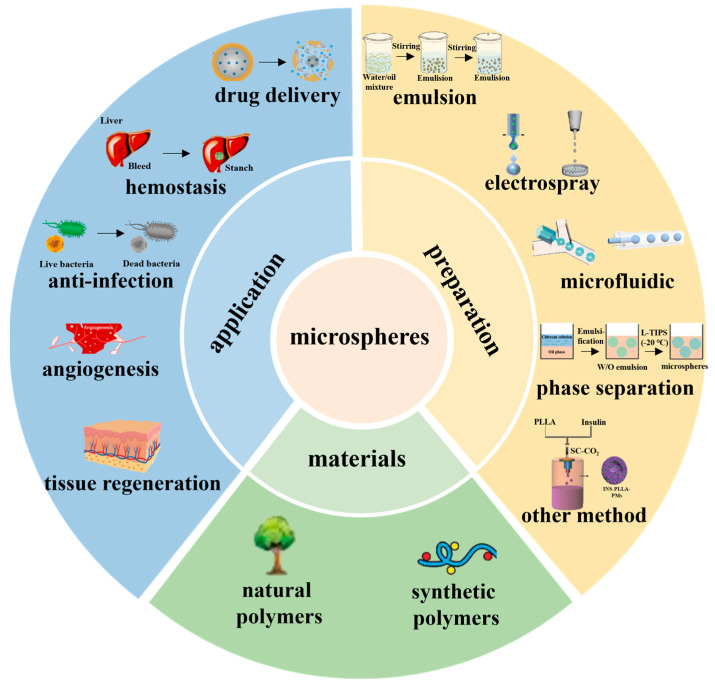
The diagram of microspheres in regard to the preparation, materials, and application.

In this review, we first briefly describe the wound healing process and simply introduce the stages of wound healing. Next, we discuss the methods of preparation of multifunctional microspheres as well as the different raw materials for preparing microspheres. Then, we also further discuss application of the microsphere as skin wound dressings in wound healing and other applications such as tumor treatment, cell culture, bone tissue engineering (Figure 1). Finally, we analyze the potential opportunities and limitations.

## 2. The Process of Wound Healing

**Figure 2:**
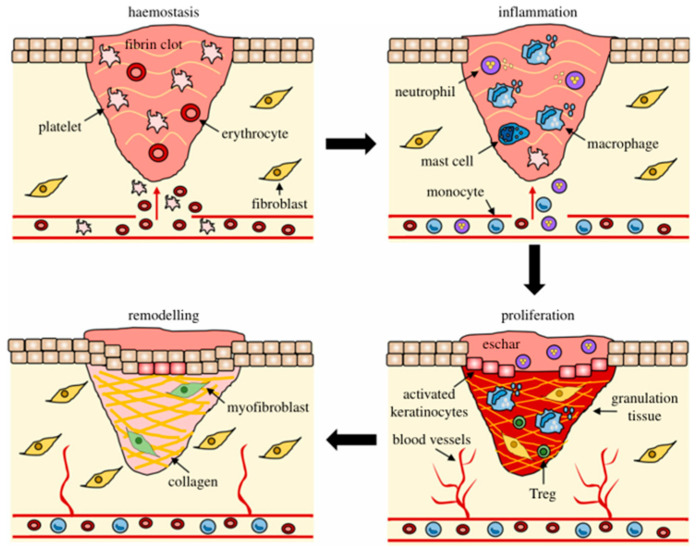
The different stages of wound healing [31]. Copyright 2020, The Royal Society of Chemistry. The stages of wound healing include hemostasis, inflammation, proliferation, and remodeling. After the skin injury, hemostasis begins. Platelets play a role in preventing blood loss. Then, neutrophils and monocytes play a role in preventing infection during the inflammation. The proliferation phase includes the formation of blood vessels and granulation tissue. Finally, myofibroblast further remodels the matrix during remodeling phase.

Typically, wound healing consists of four successive and overlapping phases: hemostasis, inflammation, proliferation and remodeling (Figure 2) [10,11]. After the skin injury, the hemostatic phase begins immediately. This phase mainly involves vasoconstriction and coagulation waterfall-like reaction to initiate hemostasis [18]. Platelets are main contributors in the process of the hemostasis and coagulation. At the same time, clot formed by platelet activation can release a variety of growth factors, such as epidermal growth factor (EGF), platelet-derived growth factor (PDGF), insulin-like growth factor 1 (IGF-1), and transforming growth factor-β (TGF-β), which diffuse into surrounding tissues and recruit inflammatory cells to remove the injury factors (such as foreign bodies) and necrotic tissues at the wound site to prevent infection [19,20]. During the inflammation, neutrophils and monocytes can also prevent infection by a series of responses. The proliferation phase mainly consists of the formation of granulation tissue and the onset of vascularization [21,22]. The remodeling phase is also referred to as the maturation phase. As the final stage of wound healing, the duration of the maturation phase varies from 3 weeks to 2 years after injury [23,24]. Myofibroblast further remodels the matrix in this process.

The most common pathological change in wound healing is chronic wounds. Chronic wounds are wounds that do not heal in the established sequence and steps of normal wound healing [25]. Many pathophysiological factors are contributed to the failure of the normal wound healing process, including systemic factors (e.g., inflammation, malnutrition, age, diabetes, etc.) and local factors (e.g., infection, tissue maceration, etc.) [2,26]. Chronic wounds are often characterized by a prolonged period of inflammatory response, delayed cell proliferation, less angiogenesis, and inadequate re-epithelialization and remodeling [27]. Treatment of chronic wounds mainly consists in preserving the moist of wound, controlling infection, and removing necrotic tissue [28]. The most common type of chronic wound is caused by elevated levels of MMPs and proteases secreted by inflammatory cells leaded to insufficient cytokines at the wound site, especially for necessary growth factors during the wound closure [29,30]. In response to these characteristics, the microspheres prepared using different materials and preparation methods are widely used in wound healing, which will be described in detail in later sections.

## 3. Preparation of Microspheres

There are various preparation methods of microspheres, and these methods have their specific characteristics. At present, commonly used methods include emulsification method, electrospray technology, microfluidic technology, phase separation method, etc. (Table 1). According to the nature of the drug and the purpose of preparing microspheres, the appropriate method is selected to prepare microspheres.

### 3.1. Emulsification

The most common method for preparing microspheres is emulsification method, and it includes single emulsification as well as double emulsification method. According to the properties of encapsulated drug, the appropriate method of preparation of microspheres is chosen.

#### 3.1.1. Single Emulsification

**Figure 3:**
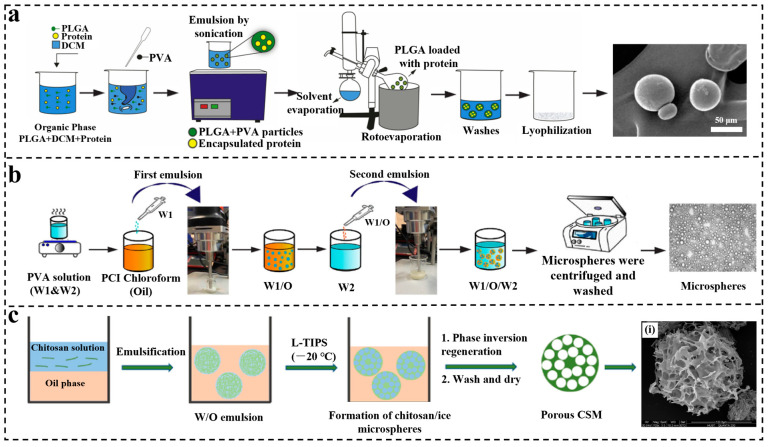
The different emulsion and phase separation methods. (**a**) Schematic of the fabrication of microspheres using single emulsion [47]. Copyright 2019, Elsevier B.V. All rights reserved. (**b**) Schematic of the fabrication of microspheres using double emulsion (W1/O/W2) [48]. Copyright 2022, Elsevier B.V. All rights reserved. (**c**) Schematic of the fabrication of the microspheres using the phase separation method and (i) SEM image of prepared microsphere. [35]. Copyright 2018, Elsevier Ltd.

Single emulsification is mainly used to encapsulate the hydrophobic drugs or poorly hydrophilic drugs. Specific preparation method of single emulsification: the drugs as well as a polymer solution can be dissolved into volatile organic solvent to obtain the mixture, and then the prepared mixture is dispersed in an aqueous phase containing an emulsifier. The aqueous phase is incompatible with the mixture. Finally, the two phases are continuously stirred to evaporate the organic solvent, and then solid microspheres are obtained [32] (Figure 3a). Stavroula Nanaki et al. used single emulsification method to prepare copolymer microparticles for long acting injectables of Naltrexone drugs [42]. This method is relatively simple to prepare microspheres, and the diameter distribution of obtained microspheres is not uniform.

#### 3.1.2. Double Emulsification

The double emulsification method can be seen as an improved, based on the single emulsification method, which is used to encapsulate the hydrophilic drugs that are difficult to dissolve in organic solvents. This method includes the water-in-oil-in-water (W1/O/W2), solid-in-oil-water (S/O/W), and water-in-oil-in-oil (W/O1/O2) emulsification method [43]. Among them, specific preparation method of W1/O/W2 emulsification is as follows: the drug is first dissolved in water, and then a polymer is dissolved in organic solvents, and then both are stirred at high speed and emulsify to form W1/O droplets, and W1/O droplets are finally placed in the external water phase (W2) containing an emulsifier to further emulsify to form W1/O/W2 droplets [33] (Figure 3b). The method is simple to operate and easy to control the parameters of process and does not require pH adjustment or temperature changes [44]. However, due to the poor lipophilicity of the encapsulated drugs, the microspheres generated with this method have drugs loading capacity (LC) as well as poor encapsulation efficiency (EE). Some research has shown that many strategies can be effective in improving the EE of the microspheres, such as using the solid particles of the drugs, improving the lipophilicity of the drug, and adding stabilizers in the W2 [45]. In addition, in the W/O1/O2 emulsification, the mixture of polymer carrier solution as well as drug aqueous solution is added in an oil phase, and the polymer carrier solution is immiscible with this oil phase [46]. This W/O/O emulsification method can effectively decrease the leaking of drugs into the outside water phase (W2).

### 3.2. Phase Separation

Phase separation method is also known as a solvent/non-solvent method. Its preparation method is as follows: emulsion (or suspension) can first be obtained by mixing the drug and carrier polymer, and then adding another inorganic salt or non-solvent substance to the emulsion (or suspension) to reduce the solubility of the drug and the polymer. Therefore, the polymer condenses from the mixture solution and precipitates on the surface of the drug to form a protective layer, and the microspheres are obtained after the protective layer is cured [34]. The main advantages of this method are simple equipment, wide range of polymer materials, and suitable for encapsulation of various drugs. The disadvantages are easily triggered adhesion and aggregation of microspheres, and the conditions are difficult to control during the fabrication process of the microspheres. Lixia Huang et al. prepared porous chitosan (CS) microspheres (CSM) through low temperature thermally induced phase separation method (L-TIPS) and micro-emulsification [35] (Figure 3c).

### 3.3. Electrospray Technology

**Figure 4:**
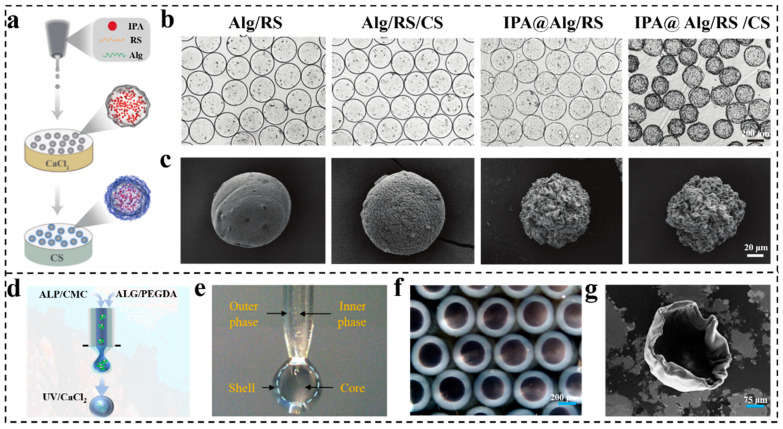
The fabrication of the microspheres using electrospray technology. (**a**) Schematic illustration of the fabrication of the microspheres using MES. (**b**,**c**) The optical (**b**) and SEM (**c**) images of the microspheres (Alg/RS, Alg/RS/CS, IPA@ Alg/RS, IPA@Alg/RS/CS) [54]. Copyright 2022, The Authors. Advanced Science published by Wiley-VCH GmbH. (**d**) Schematic illustration of the fabrication of the core-shell microspheres using CES. (**e**) The real-time picture of electrospray process. (**f**–**g**) The optical (**f**) and SEM (**g**) images of the core-shell microspheres [55]. Copyright 2020, (The Author/The Authors).

Electrospray technology, an electrostatically driven microfluidic device, has been widely used to prepare polymer microspheres and composite microspheres [36,49]. Electrospray device is mainly formed by a collection substrate, metal nozzle, syringe pump and high-voltage power supply [37,50]. The precursor solution can be injected in metal nozzle via the syringe pump and forms the specific “Taylor cone” under with the help of the high-pressure difference formed between the metal nozzle and the collection substrate [51,52]. The size of the prepared droplets is between one hundred and several hundred microns, and the solution concentration, voltage, and flow rate can affect the diameter. Specifically, size of the droplets increases with the increase in solution concentration and flow rate and decreases with the increase in voltage [53]. Electrospray technology includes mono-Coaxial ES (MES), coaxial ES (CES) and multiplexed ES (MCES). MES is traditional electrospray method, which consists of a single needle. Keli Yang et al. prepared the alginate (Alg) and resistant starch (RS) microspheres by MES which could encapsule drug and be coated by CS [54] (Figure 4a–c). CES is formed by the outer needle as well as the inner needle. In general, the polymer solution and filler are injected in the outer and inner needle through two syringe pumps, and two solutions are broken down into multilayer droplets at the nozzle outlet under the high-voltage electric field. This method can be mainly used to encapsulate proteins or drugs. In addition, the CES is also used to prepare the core-shell microspheres [55] (Figure 4d–g). MCES is comprised of several parallel capillaries, which are mainly used for large-scale production and increase the yield of microspheres [56]. Compared with the traditional method, an even greater advantage of electrospray technology is that it can prepare high-purity microspheres and be suitable for various polymers, and its operation is relatively simple. In addition, it is often used to encapsulate the sensitive substances, such as cells and proteins.

### 3.4. Microfluidic Technology

**Figure 5:**
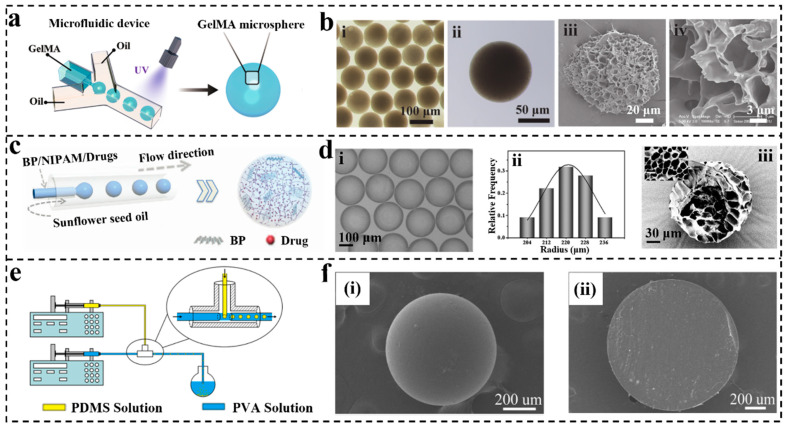
The fabrication of the microspheres using the microfluidic technology. (**a**) Schematic of the fabrication of the GelMA microspheres using microfluidic technology of the device of flow-focusing channel. (**b**) Optical (i,ii) and SEM (iii,iv) images of GelMA microspheres [60]. Copyright 2022, Wiley-VCH GmbH. (**c**) Schematic of the fabrication of the PDDMs microspheres using microfluidic technology of the device of co-flow channel. (**d**) Optical image (i), diameter distribution (ii) and SEM (iii) images of the PDDMs microspheres [61]. Copyright 2021, Wiley-VCH GmbH. (**e**) Schematic of the fabrication of the PDMS microspheres using microfluidic technology of the device of T-junction channels. (**f**) SEM images of outer surfaces (i) and inter surfaces (ii) of the PDMS microspheres [62]. Copyright 2022, American Chemical Society.

Microfluidic technology is the technology involved in systems that use micropipes to dispose of or manipulate tiny fluids, which can be used to prepare uniform droplets in the micron-level environments [38]. In this method, continuous liquids are separated to small droplets by the reaction between shear forces as well as surface tension [57]. The size of microdroplets is small and is usually at the micron or nanometer level, and it is influenced by the channel diameter and flow rate of the different phases [58]. Microfluidic devices are often referred to as microfluidic chips with different channel shapes, including T-connected channels, co-fluidic channels, and flow-focused channels [59]. In the flow-focused channel, the continuous phase flows in from the channels on both sides and further squeezes the dispersed phase to form discrete droplets under the shear forces. Kaili Chen et al. prepared the adhesive and injectable hydrogel microspheres by flow-focusing channel [60] (Figure 5a,b). In the co-flow channel, the dispersed phase is injected into the continuous phase through a conical capillary; the surfactants exist in the continuous phase, and the two phases have the same direction; stable droplets can be formed at the tip of the conical capillary orifice. Lu Fan et al. prepared the microcarrier including photo-responsive drug delivery microspheres (PDDMs) generated by the microfluidic technology of the device of co-flow channel [61] (Figure 5c,d). In the T-junction channel, the two phases each flow through a channel and are perpendicular to each other, and the dispersed phase at the junction of the two microchannels is affected by the shear forces of the continuous phase, which causes changes and instability in the flow rate and results in the formation of droplets. This device is relatively simple to prepare and is mainly used for cell screening and DNA analysis. Zhiguang Zhang et al. prepared the polydimethylsiloxane (PDMS) microspheres by the microfluidic technology of the device of T-junction channel [62] (Figure 5e,f). Compared with traditional technology, microfluidic technology possesses lots of advantages, such as low cost, high size controllability, and small droplet volume [39,63]. For adhering to the uniqueness of microdroplets, specific microfluidic devices are further created based on the structure of microspheres as well as their functional needs [64].

### 3.5. Other Methods

Other methods are also commonly used to prepare microspheres, such as template synthesis method and supercritical fluids method [65,66]. The hollow microspheres can be prepared by the template synthesis method. The template synthesis method is based on the template particles forming a polymer shell, and then removing the template to obtain microspheres with a hollow structure [40]. The conditions for preparing microspheres by template synthesis method are relatively mild and pollution-free. Moreover, the biological template material is non-toxic to the human body, which is suitable for the application of food, medicine and other industries, and has a good production and application prospect [67]. The supercritical fluid method can be performed by homogeneously mixing the drug, polymer, and supercritical fluid solution, and changing the saturation state of the whole system by adjusting the temperature and pressure so that the solute precipitates from the whole system and the polymer wraps around the drug to form microspheres with uniform particle size [68].

In summary, the emulsification method is relatively simple to prepare the microspheres. However, the diameter of the microspheres prepared with the single emulsification method is not relatively uniform. The phase separation method is suitable for multiple polymer materials and encapsulation of various drugs. However, the microspheres prepared with the phase separation method are easy to adhere and aggregate, and the conditions of this prepared process are difficult to control. For the electrospray technology, its operation is relatively simple, and the high-purity microspheres can be prepared with this technology. However, the microsphere size is affected by many electrospray parameters. The microfluidic technology has the advantages of low cost, high size controllability, and small droplet volume, but the requirements are high for the microfluidic devices. The synthesis conditions of the template synthesis method are relatively mild and pollution-free. The supercritical fluids method has good process reproducibility, but the microspheres prepared with this method may adhere to the inside of the spray dryer and cause material loss.

## 4. The Material for Microspheres

The material used in the preparation of drug-loading microspheres or wound dressings should have the following characteristics: 1. It should have good biocompatibility and blood compatibility, and not cause allergic reactions. 2. It should have good binding ability with drugs or good drug-loading rate and encapsulation rate. 3. It can be compatible with drugs and should not affect the pharmacological effects of drugs. 4. It should not be toxic and irritating and should be able to increase the stability of drugs and reduce the toxicological effects of drugs. 5. It should have good water absorption and degradation properties. 6. It should have the required viscosity, permeability, and solubility. At present, the materials used for the preparation of drug-loaded microspheres or wound dressings can be divided into natural polymers and synthetic polymers (Table 2).

### 4.1. Natural Polymers

Natural polymers are mainly substances of natural origin, and they are usually highly biocompatible and biodegradable [80,81,82]. The polymers mainly include alginate (Alg), chitosan (CS), collagen, gelatin, etc.

#### 4.1.1. Alginate (Alg)

Alg is a natural polysaccharide and is alternately composed of (1,4)-linked α-L-guluronic acid (G) and of β-D-mannuronic acid (M) [81,83]. Alg is the extract commonly obtained from seaweed [84]. Alg is a natural polymer compound, which is highly viscous and rich in hydroxyl, carboxyl, and other strong hydrophilic groups [85]. One of the main properties of Alg is the strong ability to form hydrogels, which mainly crosslinks with different divalent cations (Ca^2+^, Sr^2+^, Ba^2+^, etc.) to form the 3D networks [86]. The mass fraction of Alg solution is an important factor to affect the characteristics of microspheres. The diameter of microspheres prepared by lower concentrations of Alg is uneven, and some of them exhibit the tailing phenomenon, while higher concentrations of Alg are difficult to use to prepare microspheres due to large viscosity [87,88]. In addition, the concentration of Alg solution affect the loading rate and encapsulation rate of the microspheres. When the Alg concentration was less than 4%, the drug loading rate and encapsulation rate of microspheres gradually increased, and after more than 4%, the drug loading rate and encapsulation rate decreased with the increase in Alg concentration [89].

#### 4.1.2. Chitosan (CS)

CS is a biopolymer of biological origin naturally occurring in animals and plants (such as shrimp shells), which is obtained by the deacetylation reaction of chitin, and it is the only basic polysaccharide that exists in large quantities in the biological world [90,91,92]. It has naturally excellent antibacterial properties due to its functional group (amino group) [93]. Therefore, CS microsphere dressings have good anti-inflammatory properties. However, the antimicrobial activity of CS microsphere dressings is affected by the degree of deacetylation (DD), the concentration, and pH of the CS solution. With an increase in the DD and concentration, the antibacterial capacity of CS microspheres increases. The bactericidal activity of CS solution below pH 5.0 and above pH 9.0 is mainly caused by the influence of acids and bases on bacteria. At pH 6.0, the antibacterial activity of CS solution against *E*. *coli* and *S*. *aureus* increases with the increase in DD [94,95]. CS dressings are widely used in clinical practice, which also confirms the excellent properties of CS [96,97,98].

#### 4.1.3. Collagen

Collagen is the main component of mammalian tissues such as skin and tendons [99]. In general, chemical crosslinking methods are used to crosslink collagen. The common crosslinking reagents of collagen include formaldehyde, glutaraldehyde and carbodiimide [100,101,102]. When collagen microspheres are prepared by water-in-oil-in-water double emulsion solvent evaporation technology, the microspheres with different diameters, morphology and encapsulation rate can be prepared according to different collagen concentration and emulsification times. The higher the percentage of collagen, the longer the drug release curve [103,104,105]. Although there are many studies on collagen materials, there are not many clinical applications.

#### 4.1.4. Gelatin

Gelatin is commonly used protein biodegradable material [106]. Gelatin is a water-soluble biodegradable polymer that belongs to the hydrolysate of collagen, and it has a variety of bioactive motifs including arginine–glycine–aspartate (RGD) [107,108]. Generally, gelatin microspheres are prepared by the chemical crosslinking method, and glutaraldehyde is a common reagent for protein crosslinking, which can be used for the crosslinking preparation of gelatin microspheres [109,110]. Gelatin solution concentration is one of the vital parameters to affect the diameter and morphology of microspheres. Excessively low gelatin solution concentration is difficult to use to form microspheres or agglomerate. By increasing the concentration of gelatin solution, the shape and dispersion of microspheres are greatly improved, and the phenomenon of mutual adhesion and agglomeration disappears. In addition, the diameter of the microspheres gradually increases with the increasing in concentration of gelatin solution [111,112,113]. GelMA is a gelatin derivative that is reacted by methacrylic anhydride with gelatin and obtained by ultraviolet crosslinking in the presence of photoinitiators [114,115]. For GelMA microspheres, the variables that affect the mechanical strength and mechanical properties of the microspheres are the degree of substitution of GelMA, the concentration of GelMA and the content of initiator, UV light intensity and UV crosslinking time. The mechanical properties of the crosslinked hydrogel microspheres can be adjusted by changing the degree of substitution and concentration of GelMA material. The higher the concentration of the configuration, the greater the hardness after curing [116,117]. 

### 4.2. Synthetic Polymers

Synthetic polymers have a longer degradation cycle than natural polymers and good mechanical strength and biocompatibility [118,119]. The polymers mainly include polyester, polyamide, and polyurethane.

#### 4.2.1. Aliphatic Polyester

Aliphatic polyesters are the most studied and widely used in the application of synthetic polymers, which can be divided into polyglycolic acid (PGA), polylactic acid (PLA), polycaprolactone (PCL) and other copolymers [120,121]. Among them, the PLA is the most popular. PLA can be generated through dehydration and polymerization of lactic acid, which has good biocompatibility and biodegradability [75]. The metabolism of PLA in the body occurs through polyester hydrolysis. PLA is metabolized to carbon dioxide and water that is excreted from the body and does not accumulate in important organs [122]. PLA has been widely used in medical surgical sutures and materials for injection into microcapsules, microspheres, and implants [123,124]. The influence of PLA molecular weight on the diameter distribution of microspheres is not obvious. However, the molecular weight of PLA affects the encapsulation rate of PLA microspheres to drugs. The encapsulation rate of drugs increases with the increase in PLA molecular weight. In addition, the microspheres are stable at 4 °C and room temperature. However, under the condition of 37 °C, the adhesion aggregation occurs between the microspheres due to the softening of PLA. PLA of suitable molecular weight should be selected according to the purpose of the experiment to obtain microspheres of the desired properties [125,126,127].

#### 4.2.2. Poly(orthoesters)

Poly(orthoesters) (POE) is a class of synthetic polymers. The degradation and drug release behavior of the microspheres prepared by POE can be controlled by the addition of acidic or basic excipients [78,128]. Some POE are prepared by ester transfer reaction between diethoxytetrahydrofuran and dialcohol, which is easily degraded under acidic conditions [129]. Therefore, the sodium carbonate (Na_2_CO_3_) is added to control its degradation. Some other POE cannot produce acids during degradation. Therefore, the degradation rate does not increase due to autocatalysis caused by acids produced during the degradation process [130]. Different POE materials or microspheres can be synthesized by combining different polymers or different feed molar ratios, so that microspheres have rich physical properties and different water solubility, biocompatibility, and degradability [131].

#### 4.2.3. Polycarbonate

Polycarbonate compounds with excellent biocompatibility biodegradable ability have been widely applied in the fields of surgical sutures, drug release carriers, and tissue engineering [132,133,134]. Polycarbonate is amorphous and cannot crystallize independently to form polymer microspheres. Microspheres are formed by emulsification with other polymers or aqueous phases. In this process, the formation of microspheres is affected by the water–oil ratio and the concentration of each polymer [135]. Bin Hu et al. prepared magnetic polymer microspheres loaded with Fe_3_O_4_ magnetic ultrafine powder and tumor-targeted conjugate T9-TNF through solvent evaporation method using poly (trimethylene carbonate-co-5,5-dimethyltrimethylene carbonate) (PC, P(TMC-co-DTC)) as polymer carrier. The magnetic polymer microspheres had strong magnetic responsiveness, drug loading rate and stable drug release rate [136].

## 5. Applications in Wound Healing

The wound healing of skin is a complex process which replaces damaged cells with new, healthy cells and repairs damaged tissue structures [137]. After skin damage, the body rapidly re-establishes the skin barrier function through a series of immune responses [138]. However, due to some local factors such as wound contamination, the wound healing tends to develop into the chronic wounds and takes longer to repair [139]. As a novel wound dressing technique, microspheres can promote tissue remodeling with ability of delivering drugs, hemostasis, anti-inflammation, and promoting angiogenesis and collagen deposition at the wound site [140,141,142].

### 5.1. Drug Delivery

Conventional administration methods such as oral administration and injection often require multiple doses a day, which is not only inconvenient to use, but also causes blood levels to fluctuate widely [143]. Therefore, extended and controlled release formulations that provide durable drug release and reduce the frequency of dosing have been extensively investigated [144]. Extended-release formulations refer to formulations that can continuously release the drugs after administration to achieve the purpose of prolonging the efficacy of the drug [145,146]. It has many advantages: 1. For drugs with short half-lives or frequent administration, the number of doses can be reduced, the compliance of patients with medications can be greatly improved; 2. It can stabilize the blood concentration, which is conducive to reducing the toxic side effects of drugs; 3. It can reduce the total dose of medication.

**Figure 6:**
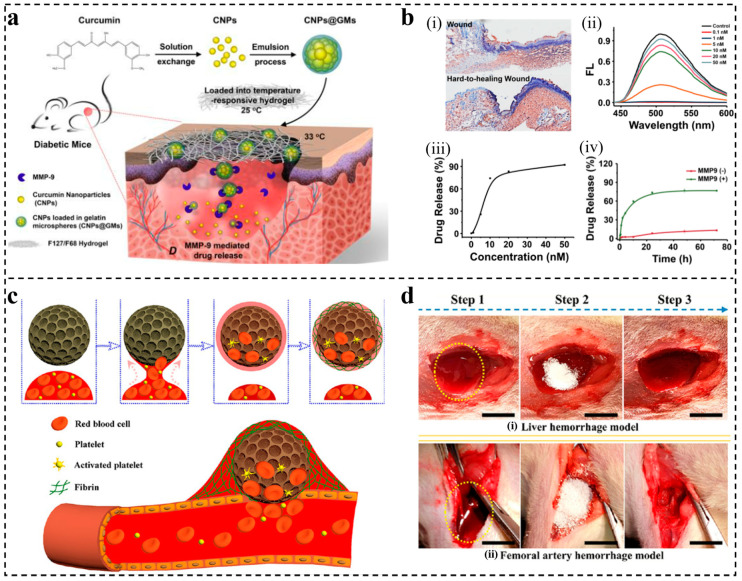
The application of the drug delivery and hemostatic of the microspheres. (**a**) Schematic of the drug release from the CNPs@GMs on the wound bed. (**b**) Enzyme-responsive drug release from CNPs@GMs. (i) MMP9 expression in the wound of mice. (ii) The fluorescence change in Cur as the concentration of MMP9. (iii) Cur released curve from microspheres with MMP9 addition. (iv) Time course of Cur release [150]. Copyright 2018 American Chemical Society. (**c**) Schematic of PHM_4_ absorbing the liquid from oozing blood. (**d**) Images of liver hemorrhage model (i) and femoral artery hemorrhage model (ii) after treatment with different method. [163]. Copyright 2019, American Chemical Society.

At present, numerous studies have developed a variety of different microsphere sustained release systems for acute and chronic wound healing [147,148]. Microspheres are suitable for the preparation of various forms of wound dressing formulations due to their small size (1–1000 μm), transmission environmental response characteristics, bio-adhesion and swelling. Microspheres can also be prepared by a variety of different techniques, as well as by selecting different polymeric raw materials for different needs. Therefore, it is considered to be a promising dressing that can better repair the skin. Drugs commonly encapsulated in the spheroid sustained release system and used for wound healing include antimicrobials, growth factors, nucleic acids, and other bioactive molecules [149]. For example, Juan Liu et al. prepared the gelatin microsphere (GM) loaded curcumin (Cur) nanoparticles (CNPs), and they were loaded into thermos-sensitive hydrogel. Cur can release from the microspheres on the wound site. The CNPs can overexpress the matrix metalloproteinases (MMPs) [150] (Figure 6a,b).

After wound formation, microorganisms such as bacteria or fungi can easily enter the wound to form infection and further delay wound healing. Antibiotics or antimicrobials such as minocycline, vancomycin, and norfloxacin are often encapsulated in microspheres to treat infectious wounds, and the maximum efficacy is usually achieved at lower concentrations [151,152]. However, long-term use can lead to the development of multidrug-resistant bacteria. Therefore, some broad-spectrum antibacterial drugs such as metal ions, metal nanoparticles (silver, copper, zinc, etc.) or antibacterial polypeptides have been discovered and applied [153,154].

Growth factors are often encapsulated in microspheres due to their short half-lives and high price [155], which not only avoids the waste caused by direct application to the affected area, but also exhibits controlled and sustained release, thereby regulating and controlling wound healing. Effective and commonly used growth factors include VEGF as well as platelet-derived growth factor (PDGF). Jinjiang Huang et al. prepared an adjustable drug delivery system including PLGA microspheres. Among them, vancomycin is attached to an injectable hydrogel, and VEGF is encapsulated into the PLGA microspheres, which not only effectively inhibits bacterial growth, but also accelerates the proliferation of venous endothelial cells [156].

### 5.2. Hemostasis

The hemostatic phase is the initial stage of wound healing. The hemostatic phase instantly presents during skin damage, but uncontrolled bleeding remains the leading cause of early post-traumatic death [157]. It is important to design biomedical hemostatic materials that can degrade in living organisms, stop bleeding rapidly, have good biocompatibility, and facilitate wound healing. Currently, the main medical hemostatic materials include patches, CS and collagen [158]. Microspheres are widely used as wound hemostatic dressings because of their strong adaptability to irregular wound shape and rich natural source materials [159]. Microspheres can be loaded with drugs such as bletilla striata polysaccharide (Bsp) to further promote hemostasis [160]. Xiaowei Wu et al. developed a compact calcium alginate shell coated with porous CS nuclear layer microsphere hemostatic agent [161]. Similar materials have been used in hemostasis [162]. Guanghui Xi et al. reported a polysaccharide hemostatic microsphere, which has a unique large pore on the surface of the microsphere. It shows high water absorption and can guide blood into microsphere [163] (Figure 6c,d). The results showed the rapid hemostasis ability of the microspheres caused by absorption of the red blood cells and platelets and forming fibrin.

### 5.3. Anti-Infection

**Figure 7:**
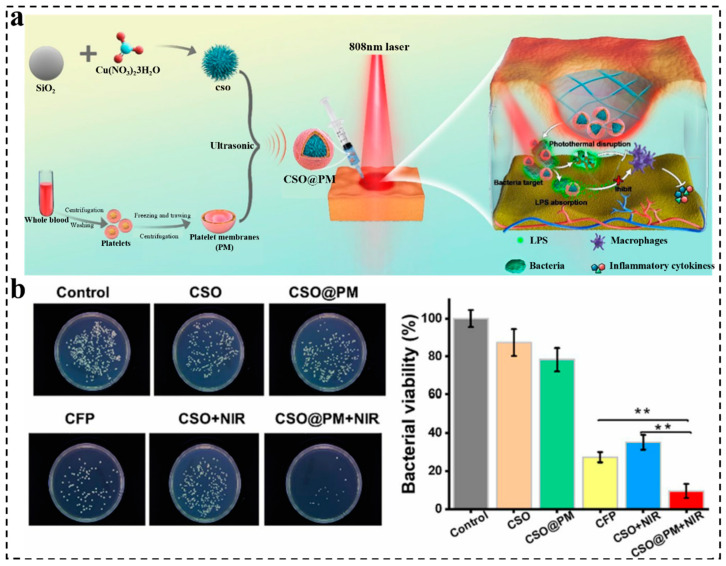
The application of the anti-infection of the microspheres. (**a**) Schematic of the preparation and application of the CSO@PM microspheres. (**b**) The photographs and counts of *P.aeruginosa* colonies from the wound sites [170]. ** indicates *p* < 0.001 compared with control group. Copyright 2021, the author(s).

In clinical practice, non-healing wounds caused by different types of skin tissue defects can bring serious economic burden and health hazards to patients, and severe skin defects can even threaten people’s lives [164]. In addition, the natural skin barrier is destroyed, and the damaged site is vulnerable to the invasion of microorganisms in the external environment, so many patients with open wounds are affected by wound infection, which is easy to cause severe wound inflammation and even secondary death [165]. Therefore, wound protective dressings with anti-infective effects are of great significance. As a new type of wound dressing, the microspheres are also constantly updated and gradually develop from a single-function dressing to a multifunctional dressing. CS is the most used natural antibacterial material [166]. To enhance the antibacterial effect of the microsphere dressings, antibiotics or antibacterial agents such as vancomycin are usually introduced [167]. Xiaoling Yu et al. developed a porous and pH-responsive polylactic acid-glycolic acid (PLGA)-vancomycin (VAN) microsphere. In a weakly acidic environment, the drug is released from the microspheres, and the microspheres show potent antibacterial activity [168]. However, long-term use of antibiotics can lead to the development of multidrug-resistant bacteria. Therefore, antimicrobial peptides or metal nanoparticles are often introduced into the microspheres, which not only improve the antimicrobial effect, but also prevent the development of multidrug-resistant bacteria [169]. Furthermore, photothermal antibacterial platform is also often introduced into the microspheres and has good antibacterial effect. For example, Zaihui Peng et al. prepared a novel antibacterial platform of platelet membrane-coated copper silicate hollow microspheres (CSO@PM). It is non-toxic and has highly effective antibacterial properties [170] (Figure 7).

### 5.4. Angiogenesis

**Figure 8:**
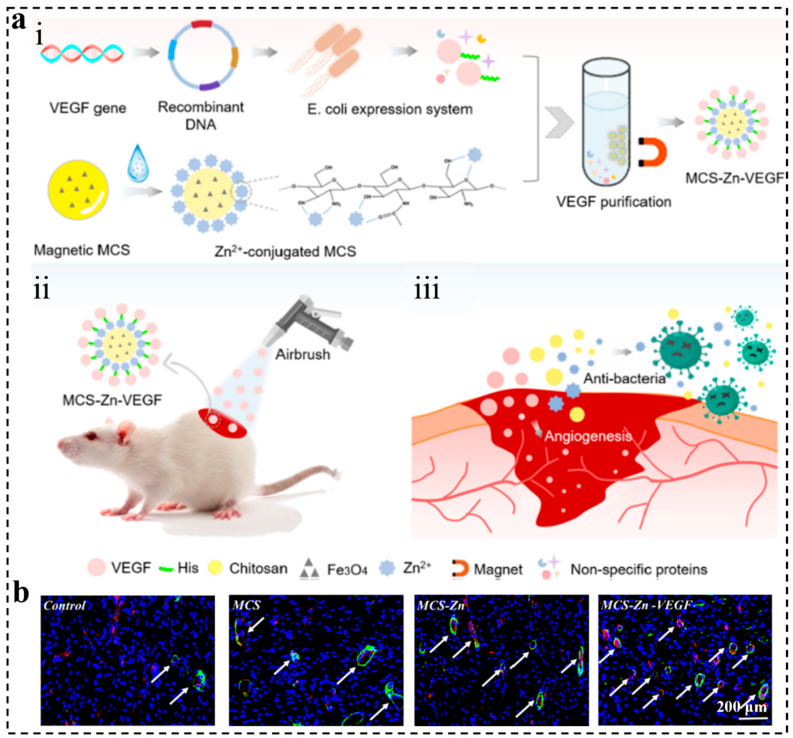
The application of the angiogenesis of the microspheres. (**a**) Schematic of the preparation and application of the MCS-Zn^2+^-VEGF microspheres. (i) Production of the recombinant VEGF and microsphere. (ii) The microspheres were used in the wound healing. (iii) The angiogenesis ability of the microspheres in the process of wound healing. (**b**) Angiogenesis ability of the MCS-Zn^2+^-VEGF microspheres used to treat the wound [177]. Copyright 2022, the Authors. Published by Elsevier Ltd.

During wound healing, angiogenesis is essential for tissue development and maturation. Angiogenesis is primarily the process of existing blood vessels growing to new capillaries or by prolonged vascular loops already existing in the body [171,172,173]. An important cause of the difficulty of the chronic wound repair is poor local blood supply. Endothelial progenitor cells can secrete a variety of vascular factors to promote the repair and regeneration of blood vessels, and meanwhile they participate in the regeneration process and have critical function in wound healing [174,175]. The application of growth factors in regard to the wound closure has been widely discussed in recent years. VEGF and bFGF are closely related to the vascular endothelial cell function [176]. At present, some previous work uses the microspheres encapsulated growth factor for wound healing [159]. Lanjie Lei et al. reported a novel multifunctional CS microsphere (MCS-Zn^2+^-VEGF), and VEGF is applied to wounds in combination with MCS. It was determined that the microspheres could not only deliver growth factors, but also exhibit the good angiogenesis ability [177] (Figure 8).

### 5.5. Tissue Regeneration

**Figure 9:**
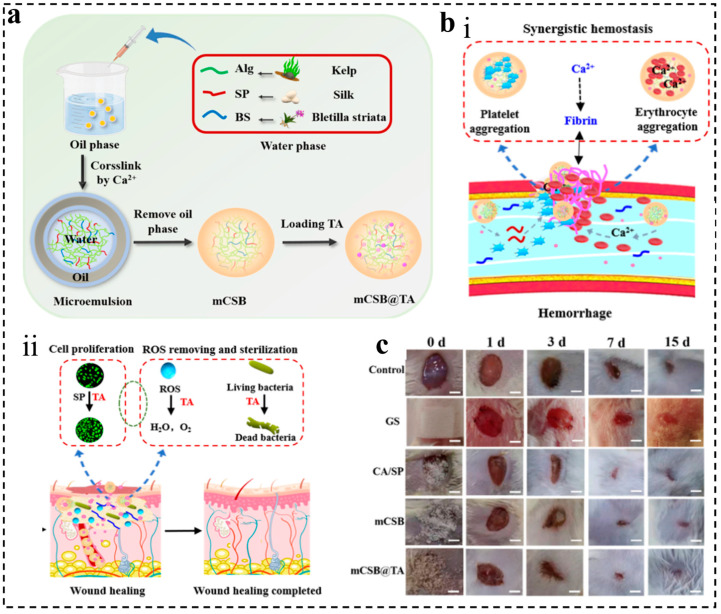
The application of the tissue regeneration of the microspheres. (**a**) Schematic of the preparation of the mCSB@TA microspheres. (**b**) Schematic of the application of the mCSB@TA microspheres for wound healing. (i) The schematic of synergistic hemostasis of the microspheres. (ii) The schematic of wound healing process. (**c**) The healing process of the wound treated with different microspheres [160]. Copyright 2022, published by Elsevier Ltd.

Repairing the structure and function of tissues after injury is essential for living organisms. The damaged tissue is gradually regenerated by different tissue engineering methods. The purpose of regeneration is to allow them to acquire normal physiological activity. As a new type of injectable biomaterial, the microspheres are used to reshape the wound tissue through antibacterial, anti-inflammatory, pro-repair, and other multi-functions [178,179]. Functional microspheres have attracted increasing attention in tissue regeneration. Jaideep Banerjee et al. prepared PEGylated fibrin hydrogels loaded with silver sulfadiazine (SSD) microspheres to treat wounds. The outcomes of the wound healing are assessed by granulation tissue formation and collagen deposition [180]. SSD-CSM-FPEG could effectively reduce bacterial infection and promote new vascular formation by improving stromal remodeling and increasing the thickness of granulation tissues. In addition, Nan Wang et al. used the reverse emulsion method to prepare the composite microspheres (mCSB) that could load tannic acid (TA). It had long-lasting antibacterial properties. After 7 days of microsphere treatment, the wound healing ratio of >92.80% was obtained [160]. The composite microspheres had good wound healing effect (Figure 9).

### 5.6. Other Applications

**Figure 10:**
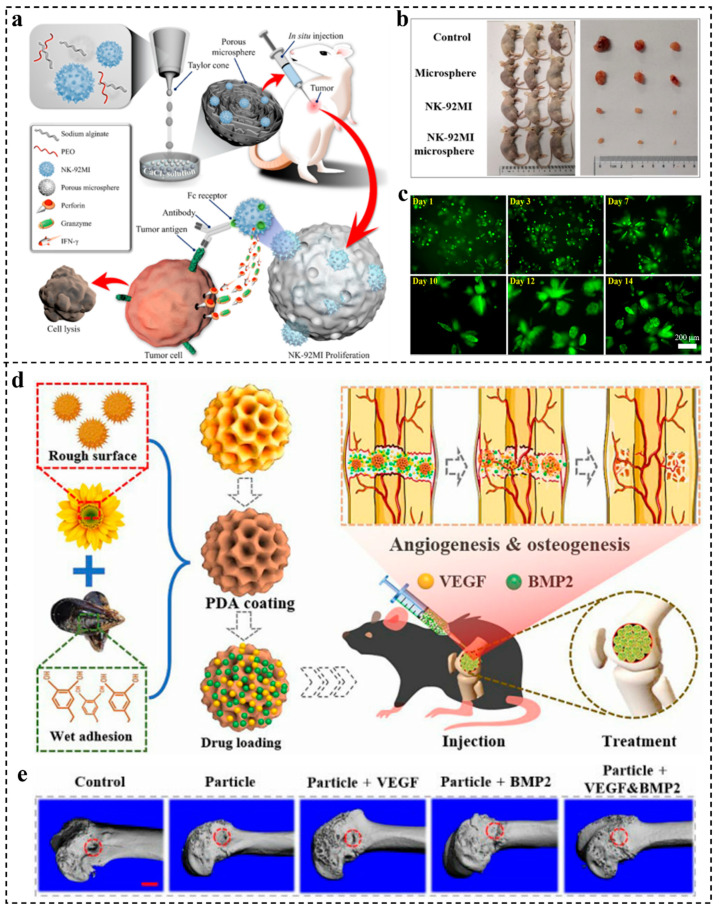
The application of the microspheres. (**a**) Schematic of the fabrication and application of the microspheres encapsulated the NK cells. (**b**) The picture of the tumor and mice treated with different method. (**c**) The images of NK-92MI cells cultured in the microspheres at different times [185]. Copyright 2019, American Chemical Society. (**d**) Schematic of the fabrication and application of the bio-inspired dual-adhesive microspheres. (**e**) The micro-CT reconstruction images after treated with the microspheres [186]. Copyright 2023, springer Nature Switzerland AG.

In addition to wound healing, microspheres are also widely used in tumor therapy, cell culture, bone tissue engineering and other fields [181,182,183,184]. Dan Wu et al. realized in situ tumor immunotherapy by preparing the microspheres encapsulating the natural killer (NK) cells. NK cells encapsulated in porous microspheres had good proliferative ability and could continuously secrete perforin and granzyme and showed powerful tumor-killing effects [185] (Figure 10a–c). Lixia Huang et al. prepared porous CS microspheres (CSM) with unique porous structure and good biocompatibility. The three-dimensional (3D) space of spheroids could be used to culture the high-performance hepatocyte [35]. Lei Yang et al. prepared a novel type of dual-adhesive hydrogel microsphere by the microfluidic electrospray. The microspheres were further coated by polydopamine (PDA). The microspheres could significantly enhance bone regeneration [186] (Figure 10d,e). Chengyu Wu et al. prepared spray-dried microparticles to research the drug delivery of leuprolide. The results indicated that the ratio of PLGA and lipids can strongly influence the release of PLGA lipid hybrid MPs [187].

## 6. Conclusions and Outlook

As mentioned above, there are different methods of preparing microspheres, and the microspheres prepared by different methods have different morphological structures and properties, and the differences in their properties make them widely used in different fields. Depending on the specific application, different preparation methods can be chosen. In addition to the diversity of preparation methods, the raw materials used to prepare microspheres are also diverse. These materials include both natural and synthetic polymers. Again, depending on the application, microspheres can be prepared from different raw materials. In addition, the development of multifunctional microspheres can be achieved by blending multiple polymers or synthesizing copolymers of two polymers to achieve multiple synergistic effects. We also describe the role of microspheres as a novel dressing for wounds or infected wounds, including drug delivery, anti-inflammatory, antibacterial and skin tissue remodeling. In conclusion, microspheres are of great significance in the field of wound healing as a new type of wound dressing.

In this review, various raw materials, preparation methods and applications of microspheres are reviewed, and this can provide a basis for research about microspheres in the future. However, the application of microspheres still faces many challenges in many fields including wound healing. To promote the development of microspheres in the field of wound repair, some disadvantages need to be addressed. For example, on the one hand, some microspheres are easy to fall off when applied to the wound surface due to poor adhesion, thus requiring multiple dressing changes to achieve better repair results. On the other hand, when the microsphere powder dressings with high water absorption are used on the wounds, the dressings can absorb inflammatory exudate on the wound site, but it is not conducive to provide a moist healing environment. In addition, good microsphere wound dressings should have a variety of characteristics such as water absorption, complying with wound shape, high drug loading rate, moisturizing, hemostasis, antibacterial and promoting healing, but most of the microspheres prepared in the current study can contain several of these characteristics, and it is difficult to contain all the excellent properties; thus, this has become a research gap in the field of microsphere wound dressing. We hope that the novel microsphere dressings with different structures and properties can be designed according to different needs in the future. Therefore, the preparation and application of multifunctional microspheres will be the research topic in the field of microsphere wound dressings in the future. We hope to see the research progress and breakthrough in the field of multifunctional microspheres in the practical clinical application soon.

## Figures and Tables

**Table 1:** The advantages as well as disadvantages of the varying methods of preparing microspheres.

Method	Advantages	Disadvantages	References
Emulsification	1. W1/O/W2 does not require adjustment of the pH and significant change in the temperature 2. Single emulsification is relatively simple	1. Waste generation 2. Use of one or more surfactants 3. Requires multiple steps 4. Low yield and high purification cost	[32,33]
Phase separation	1. Simple equipment 2. Wide range of polymer materials 3. Encapsulation of variety of drugs	1. The problems of the adhesion and aggregation of microspheres 2. The conditions are difficult to control during the process of the microspheres	[34,35]
Electrospray	1. Preparation of high-purity microspheres 2. Suitable for many types of polymers 3. The operation is relatively simple	1. In some cases, a crosslinking agent is used 2. There are many factors affecting particle size	[36,37]
Microfluidics	1. Low cost 2. High size controllability 3. Small droplet volume 4. The particle size is highly homogeneous 5. The device is relatively simple	1. Use of various solvents to remove the oil phase 2. The precision requirements of fluidic devices are high	[38,39]
Template synthesis	1. The condition is relatively mild 2. The biological template material is non-toxic to the human body	1. Templates need to be embellished 2. No template is used narrowly	[40]
Supercritical fluids	1. Good process reproducibility 2. Little effect on the stability of the drug	1. Microspheres may adhere to the inside of the spray dryer, causing material loss	[41]

**Table 2:** The molecular structure and application of the different materials for preparing microspheres.

Property	Composition	Molecular Structure	Method	Application	Reference
Natural polymers	Sodium Alginate	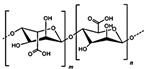	Electrospray	Wound dressing	[54,69]
	Chitosan	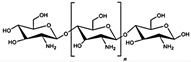	Supercritical fluids	Wound dressing	[69,70]
	Collagen	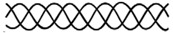	Emulsification	Skin repair	[71,72]
	Gelatin	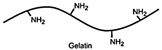	Electrospray	Packaging for food	[73]
	GelMA	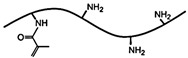	Microfluidic	Skin closure	[74]
Synthetic polymers	Polylactic acid (PLA)	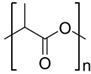	Co-solvent evaporation	Sutures	[75]
	Polyglycolic acid (PGA)	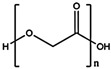	Emulsification	Surgical Suture	[76]
	PGA-PLA copolymer	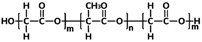	Emulsification	Implants	[77]
	Poly- (orthoesters) (PEO)	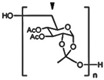	Emulsification	Drug delivery	[78]
	Polycarbonate	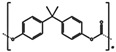	Emulsification	Drug delivery	[79]

## Data Availability

Not applicable.
